# Rapid 4D regional wall motion characterization of abdominal aortic aneurysms with intra-luminal thrombus using cine cardiac MRI

**DOI:** 10.1186/1532-429X-16-S1-P385

**Published:** 2014-01-16

**Authors:** Prahlad G Menon, Abhinav Mehra, Mark Doyle, Robert W Biederman, Ender A Finol

**Affiliations:** 1 Electrical and Computer Engineering, Sun Yat-sen University - Carnegie Mellon University Joint Institute of Engineering, Pittsburgh, Pennsylvania, USA; 2 QuantMD, LLC, Pittsburgh, Pennsylvania, USA; 3 Allegheny General Hospital, Pittsburgh, Pennsylvania, USA; 4 Biomedical Engineering, The University of Texas at San Antonio, San Antonio, Texas, USA

## Background

The clinical assessment of abdominal aortic aneurysm (AAA) rupture risk is largely limited to quantification of maximum diameter over time to monitor growth. Recent studies have extended this paradigm to modeling biomechanical loading and wall stresses using computational hemodynamics or solid mechanics simulations, in efforts to reliably numerically predict aneurysm-specific wall motion. However, the numerical simulation of aneurysm wall motion is time and resource intensive, and inherently involves complex mathematical modeling of often unrealistic wall properties. In this study, we characterize AAA wall motion using shape-analysis to quantify detailed regional function by direct processing of 4D (3D + time) cine CMR data.

## Methods

Inner blood-contacting endothelial wall (excluding thrombus) function in 13 AAAs was studied using a shape-derived metric of wall velocity defined by a signed phase-to-phase Hausdorff distance (HD) computed at uniformly spaced points tracked on surface contours segmented from CMR images, over the cardiac cycle, using an in-house software. HD colormaps were superimposed upon the AAA inner wall at the diastolic phase to visualize regional wall motion. A endothelial wall velocity (EWV: displacement in mm per phase) was estimated to be a function of phase-to-phase displacement time-histories at tracked points. Additionally, outer wall segmentations were also prepared and compared with the inner wall at each cardiac phase to quantify radial wall thickness and signed wall-thickening (diastole to systole) using an open-souce cardiac MRI suite, Medviso Segment. This was compared with the observed EWV from HD analysis for validation purposes.

## Results

**Figure 1:**
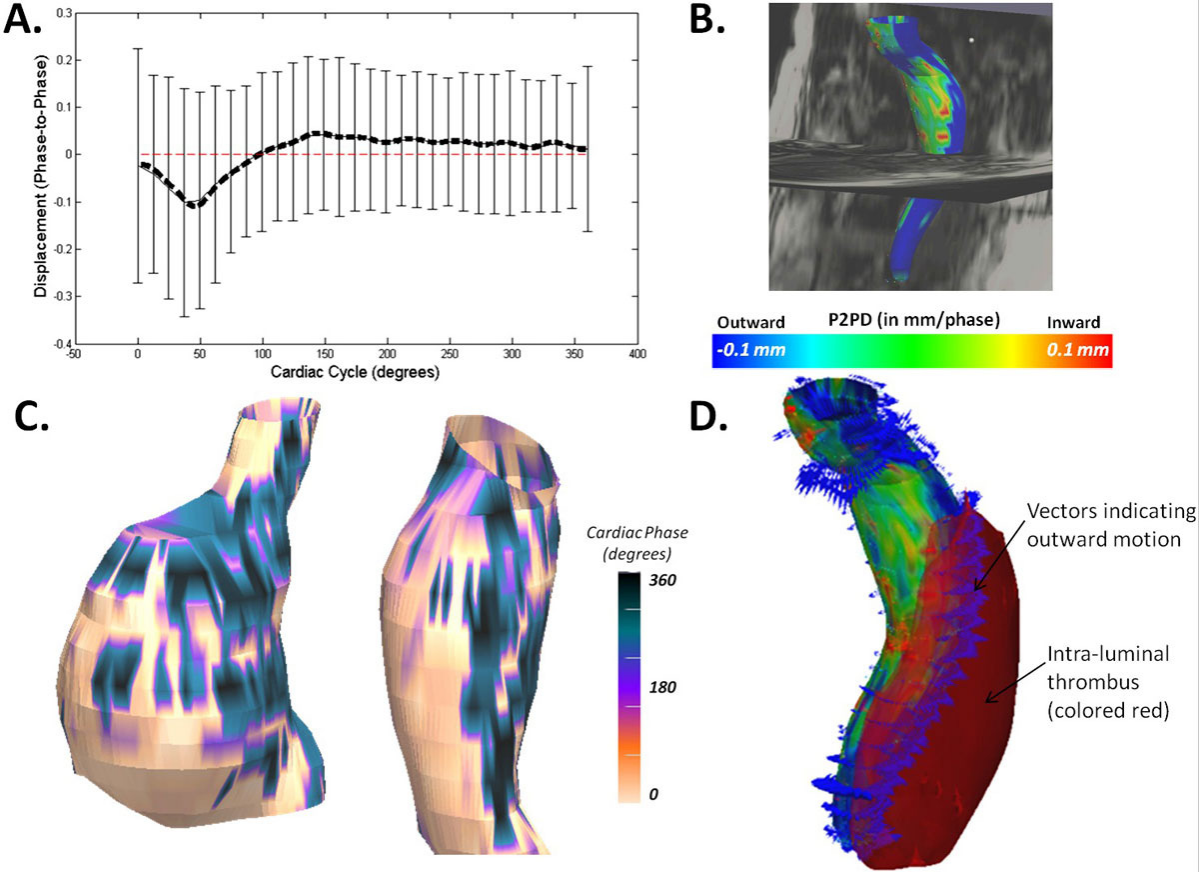
**A) Average and std dev in EWV characteristic of the AAA cohort, at each cardiac phase**. B) Colormaps of HD (in mm/phase, shown for a representative patient-specific AAA) provide a 3D instantaneous visualization of EWV at peak filling rate (expansion). C) Phase of maximum regional displacement colored on diastolic surfaces of two patient-specific AAAs indicates ILT contacting wall (white) is dyssynchronous with remaining endoluminal wall. D) Vectors indicating endoluminal wall motion, in a representative patient-specific AAA.

**Figure 2:**
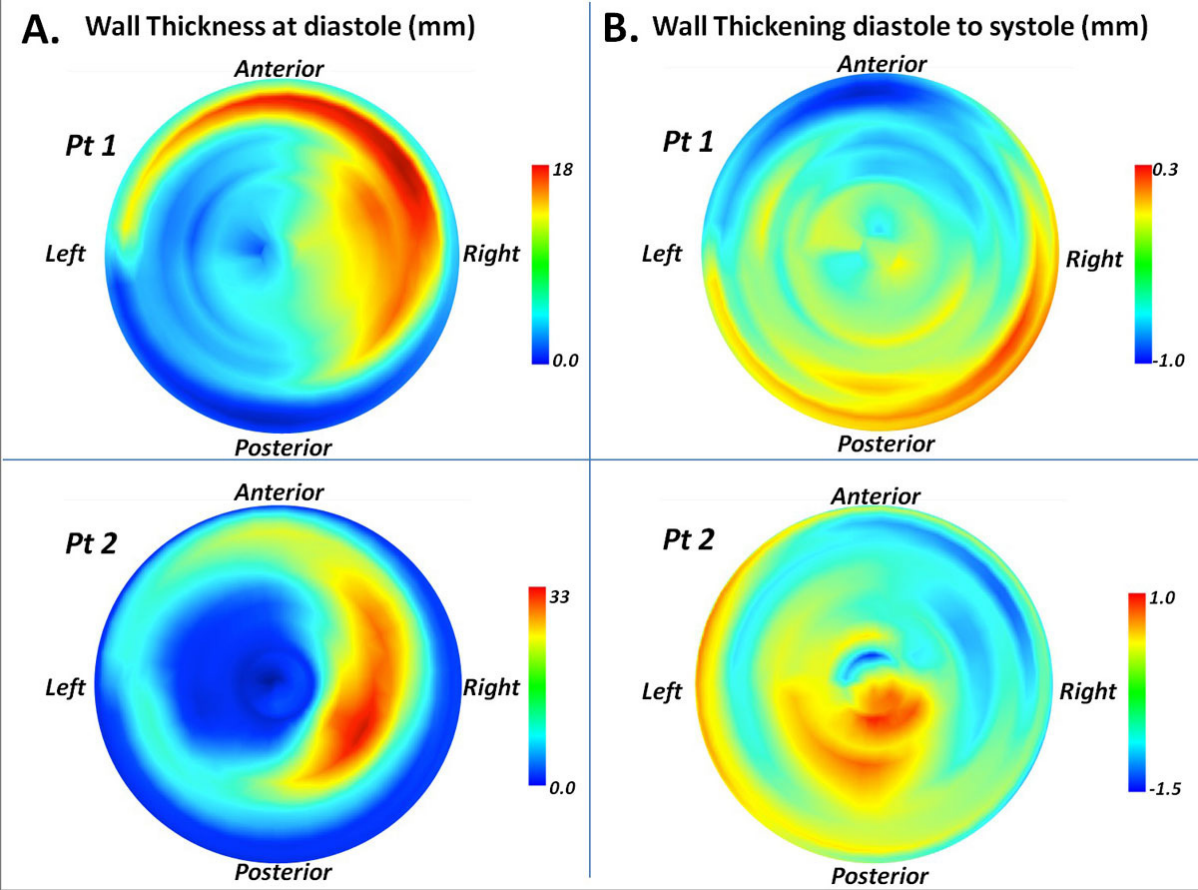
**A) Wall-thickness function polar plots (axial view) indicating thicker ILT contacting wall regions in two representative AAAs**. B) Wall-thickening magnitude was synonymous with the 4D HD analysis, providing validation of the novel method. ILT contacting wall regions were more dynamic with observable radial compression at systole and expansion at diastole.

Mean AAA volume ranged from 64.7 ± 31.6 mL to 69.1 ± 33 mL. Figure 1A shows the average and std deviation EWV characteristic of the cohort, at each cardiac phase. Colormaps of HD (Figure 1B) and phase of maximum regional displacement (Figure 1C) provided visualizations of EWV and the existence of a subtle dyssynchrony in wall motion contact with intra-luminal thrombus (ILT) as opposed to wall regions without thrombus. Outer wall motion was observed to be small in contrast with inner wall motion which had distinctly amplified EWV at ILT-contacting wall-regions (see Figure 1D). Reduction of the wall thickness and wall-thickening function maps from Medviso Segment into AHA-type segmental polar plots (see Figure 2) allowed visual correlation between ILT occupied wall regions (thicker), and regions with greater wall-thickening function (negative or wall thinning/compression at systole and positive thickening at diastole) owing to inner wall motion.

## Conclusions

We present a simple and effective means to directly quantify AAA wall motion from CMR data, without the need for numerical simulation. EWV extracted from the presented analyses may be extended to the prediction of aneurysm wall stresses and strain, which may in-turn constitute a promising paradigm for evaluating AAA rupture risk.

## Funding

This study was possible through the Open Field Entrepreneur's Fund award to QuantMD, LLC.

